# Drug-primed reinstatement of cocaine seeking in mice: increased excitability of medium-sized spiny neurons in the nucleus accumbens

**DOI:** 10.1042/AN20130015

**Published:** 2013-10-02

**Authors:** Yao-Ying Ma, Sandy M. Henley, Jeff Toll, James D. Jentsch, Christopher J. Evans, Michael S. Levine, Carlos Cepeda

**Affiliations:** *Stefan & Shirley Hatos Center for Neuropharmacology, University of California, Los Angeles, California, U.S.A.; †Department of Psychology, University of California Los Angeles, Los Angeles, CA, U.S.A; ‡Intellectual and Developmental Disabilities Research Center, Semel Institute for Neuroscience and Human Behavior, David Geffen School of Medicine, University of California, Los Angeles, CA, U.S.A.

**Keywords:** intravenous self-administration, nucleus accumbens, relapse, synaptic transmission, AHP, afterhyperpolarization, AL, active lever, BIC, bicuculline, Coc, cocaine, CsMeth, Cs-Methanesulfonate, FR, fixed ratio, GABA, γ-aminobutyric acid, IAL, inactive lever, IC, current clamp, IVSA, intravenous self-administration, KGluc, K-gluconate internal solution, mEPSCs, miniature excitatory postsynaptic currents, MSNs, medium-sized spiny neurons, NAc, nucleus accumbens, Rin, resistance, rm, repeated measure, RMP, resting membrane potential, sEPSCs, spontaneous excitatory postsynaptic currents, sIPSCs, spontaneous inhibitory postsynaptic currents, Thr, threshold, TTX, tetrodotoxin, VC, voltage clamp

## Abstract

To examine the mechanisms of drug relapse, we first established a model for cocaine IVSA (intravenous self-administration) in mice, and subsequently examined electrophysiological alterations of MSNs (medium-sized spiny neurons) in the NAc (nucleus accumbens) before and after acute application of cocaine in slices. Three groups were included: master mice trained by AL (active lever) pressings followed by IV (intravenous) cocaine delivery, yoked mice that received passive IV cocaine administration initiated by paired master mice, and saline controls. MSNs recorded in the NAc shell in master mice exhibited higher membrane input resistances but lower frequencies and smaller amplitudes of sEPSCs (spontaneous excitatory postsynaptic currents) compared with neurons recorded from saline control mice, whereas cells in the NAc core had higher sEPSCs frequencies and larger amplitudes. Furthermore, sEPSCs in MSNs of the shell compartment displayed longer decay times, suggesting that both pre- and postsynaptic mechanisms were involved. After acute re-exposure to a low-dose of cocaine *in vitro*, an AP (action potential)-dependent, persistent increase in sEPSC frequency was observed in both NAc shell and core MSNs from master, but not yoked or saline control mice. Furthermore, re-exposure to cocaine induced membrane hyperpolarization, but concomitantly increased excitability of MSNs from master mice, as evidenced by increased membrane input resistance, decreased depolarizing current to generate APs, and a more negative Thr (threshold) for firing. These data demonstrate functional differences in NAc MSNs after chronic contingent *versus* non-contingent IV cocaine administration in mice, as well as synaptic adaptations of MSNs before and after acute re-exposure to cocaine. Reversing these functional alterations in NAc could represent a rational target for the treatment of some reward-related behaviors, including drug addiction.

## INTRODUCTION

Relapse, the major challenge in the treatment of drug abuse, has been modeled in rodents by reinstatement of extinguished drug-seeking behaviors (Steketee and Kalivas, 2011). Similar to humans, this phenomenon can be precipitated by exposure to a small dose of the abused drug. Local administration of cocaine into the NAc (nucleus accumbens) reinstated drug-seeking, whereas microinjection into the dorsal striatum or lateral septum did not (Park et al., 2002), showing that the drug's effects on the behavior are anatomically specific. Using a reinstatement model, in conjunction with inactivation of different pathways involved in drug addiction, the neural circuitry involved in reinstatement of drug-seeking has been mapped (Kalivas and McFarland, 2003). In this circuit, reinstatement pathways converge onto a final common output through the NAc.

Chronic cocaine-induced functional alterations in the NAc contribute to cocaine-elicited addiction behavior, including drug-primed relapse to previously abused drugs (Hyman et al., 2006). Extensive efforts have been directed at elucidating neuroadaptations of MSNs (medium-sized spiny neurons), the principal cell type in the NAc, after chronic exposure to either non-contingent (response-independent) or contingent (response-dependent) administration of cocaine (reviewed by Wolf, 2010). MSNs in the NAc receive excitatory inputs from the pre-/infra-limbic cortex, ventral subiculum and basolateral amygdala (Voorn et al., 2004; Sesack and Grace, 2009), mixed excitatory and inhibitory neuromodulation from dopaminergic neurons and inhibitory inputs from local and afferent GABAergic circuits (Voorn et al., 2004; Yan and Nabeshima, 2009). Although there are some reports on the influence of chronic cocaine exposure on inhibitory synaptic adaptations (Meshul et al., 1998; Kushner and Unterwald, 2001; Yamaguchi et al., 2002; Frankowska et al., 2008a, b), growing evidence supports adaptions in glutamate receptor-mediated synaptic activity after chronic cocaine administration. Thus, AMPA (α-amino-3-hydroxy-5-methyl-4-isoxazole-propionic acid) receptors are up-regulated at NAc synapses after withdrawal from cocaine administration (Wolf, 2010).

The two major determinants of the functional output of NAc MSNs, i.e., synaptic transmission and intrinsic membrane excitability, have been extensively examined after cocaine administration (Dong et al., 2006; Ishikawa et al., 2009; Mu et al., 2010; Wolf, 2010). During early withdrawal (within 1 week) from either non-contingent or contingent cocaine, a decreased intrinsic excitability of MSNs has been demonstrated (Zhang et al., 1998; Zhang et al., 2002; Hu et al., 2004; Hu et al., 2005; Hu, 2007) (reviewed by Wolf, 2010). After a longer withdrawal period from cocaine exposure with a non-contingent cocaine history, a decrease in intrinsic excitability occurs in MSNs, at least in the shell compartment. However, previous studies have also suggested that the effects of contingent and non-contingent cocaine administration may differ quantitatively or qualitatively, which could limit the generality and validity of laboratory studies that use primarily non-contingent administration (Winsauer et al., 2003). Only a handful of studies have examined the functional output of NAc MSNs in the cocaine contingent administration model (Mu et al., 2010).

Considering that relapse after a long period of abstinence is a major clinical problem when treating people with a substance use disorder (Hyman et al., 2006), it is imperative to develop a reliable model of reinstatement of drug-seeking behavior. Contingent drug administration, e.g., IVSA (intravenous self-administration) in rodents, has been recognized as the best model to mimic addiction behavior in humans (Shaham et al., 2003). This model thus represents a powerful tool to explore potential mechanisms of drug addiction and the functional alterations of NAc MSNs. The present experiments were designed to study electrophysiological adaptations in NAc MSNs in the IVSA mouse model of drug-primed reinstatement using a complex paradigm that better replicates the human condition, consisting of repeated cycles of extinction-reinstatement. After establishment of cocaine-seeking behavior, *in vitro* slices from trained mice were used to examine changes in membrane excitability and synaptic transmission in two conditions: firstly, under basal conditions before cocaine re-exposure and secondly, following acute exposure to cocaine which in a way is the *in vitro* equivalent of drug-primed cocaine-seeking behavior. To the best of our knowledge, this is the first study using such drug IVSA extinction-reinstatement procedure in mice, in conjunction with examination of functional alterations in synaptic activity and excitability of NAc MSNs.

## MATERIALS AND METHODS

### Animals and housing

Male C57BL/6J mice (Jackson Laboratory, Bar Harbor, ME) were obtained at the age of 8–9 weeks and housed four per cage with food and water *ad libitum*. Ambient vivarium temperatures were maintained at ~22°C, and illumination was provided for 12 h/day (7:00 AM on). Experimental procedures were performed in accordance with the United States Public Health Service *Guide for the Care and Use of Laboratory Animals* and were approved by the Institutional Animal Care and Use Committee at the University of California, Los Angeles.

### Operant conditioning apparatus

Each operant chamber (Med Associates, St. Albans, VT) was fitted with an AL (active lever) and an IAL(inactive lever, 7 mm above the gridfloor), a cue light positioned 45 mm above the center of the two levers and a house light on the opposite wall of the chamber. The position of the AL was counterbalanced across individuals and within each group. Chambers were located in sound-attenuating containers, with a fan that always was on during the training sessions.

### Catheter implantation, maintenance and patency

We used methods previously described (Thomsen and Caine, 2005, 2007). Mice were anesthetized with isoflurane vapor mixed with oxygen and implanted with a chronic indwelling silastic catheter (0.2 mm i.d., 0.4 mm o.d.) connected to a 26-gauge guide exteriorized cannula for drug administration in the right or left jugular vein. The catheter was tunneled subcutaneously to the base located in the midscapular region. Two subcutaneous injections of carprofen (5 mg/kg) were given right before and 24 h after surgery. Mice were allowed 7 days to recover, during which 0.02 ml of 0.9% (w/v) saline containing heparin (30 USP units/ml) and antibiotic (cefazolin, 67 mg/ml) was infused daily through the catheter to forestall clotting and infection. During the following experimental procedure of cocaine IVSA acquisition, the catheter was flushed with heparinized saline before and after each training session. Catheter patency was confirmed before and after completion of cocaine IVSA acquisition phase by the infusion of propofol (0.02 ml; 10 mg/ml). Loss of muscle tone and clear signs of anesthesia within 3 s indicated catheter patency. Catheterization of rodents for IVSA, particularly mice, is technically challenging. In the present study, a total of 51 mice were used but eight were excluded due to catheter leakage or clogging (*n*=7) or surgery-associated infection (*n*=1). No mortality due to the catheterization procedure occurred.

### Behavioral procedure of drug-primed cocaine seeking

**Figure 1:**
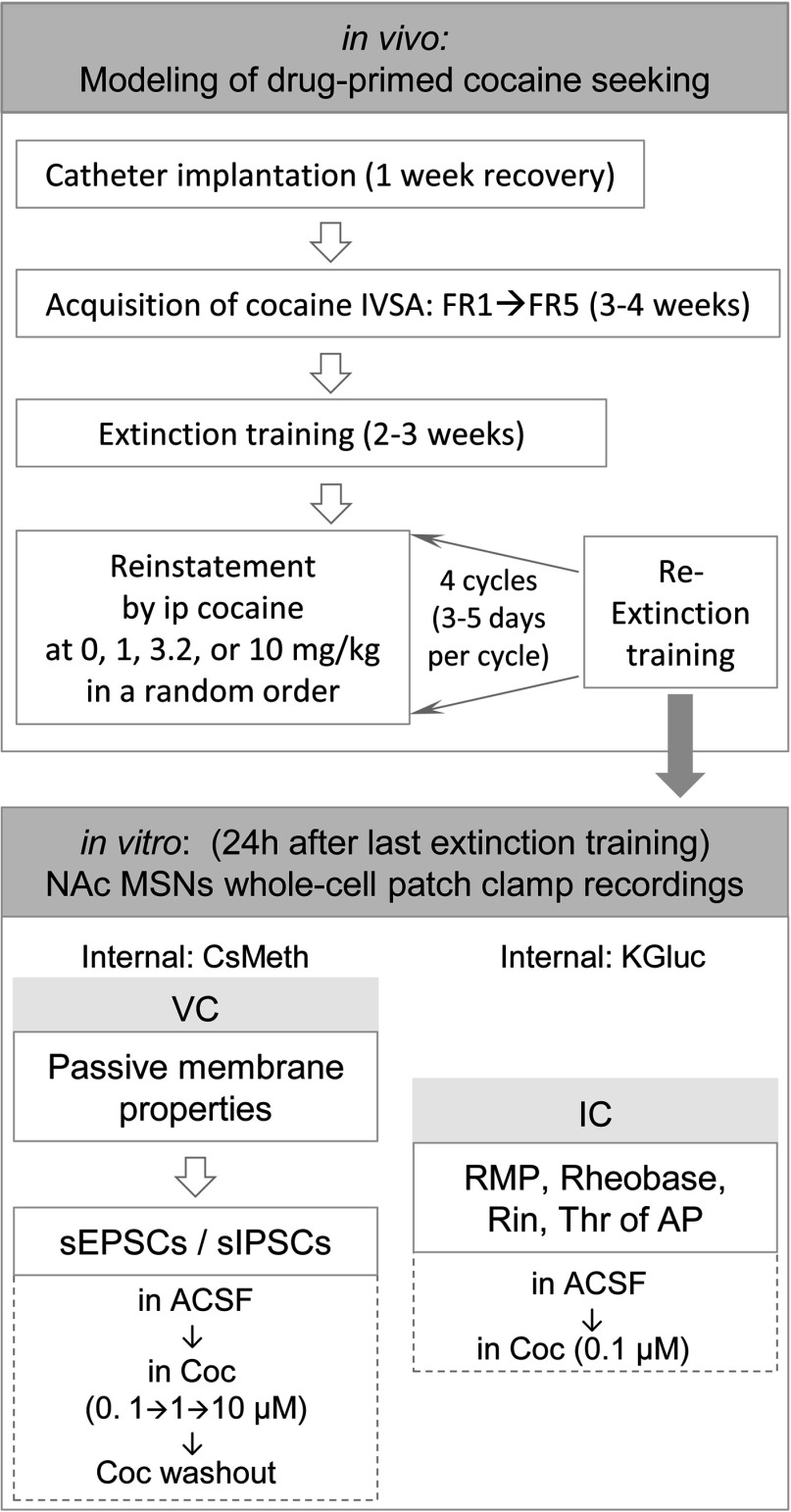
Schematic diagram of *in vivo* behavioral procedures for modeling drug-primed reinstatement of cocaine seeking and *in vitro* whole-cell patch clamp recordings of the NAc MSNs are shown in the upper and lower panels, respectively FR, fixed ratio; Coc, cocaine; IC, current clamp; VC, voltage clamp.

The schematic of *in vivo* behavioral procedure for modeling drug-primed cocaine seeking is shown in the upper panel of Figure 1. It consisted of a multi-staged procedure, which started with the acquisition of cocaine IVSA, followed by several cycles of extinction and reinstatement.

### Acquisition of cocaine IVSA behavior

One week after jugular catheter implantation, training of mice in the operant conditioning chambers was initiated. The training session lasted 2 h/day, 5 days a week for 3–4 weeks. Each session started with a house light on and both levers extended. For master mice, the training started with an FR 1 (fixed ratio 1) schedule of reinforcement, during which one response to the assigned AL resulted in the programmed consequences, i.e., an IV drug injection through the pre-implanted catheter as well as illumination of the central cue light on and the extinguishing of the house light for 20 s (time-out, no more injection was delivered after active response). The criteria for moving from FR1 to FR5 phase included: (1) earning a minimum of 20 reinforcers per session in two consecutive sessions; (2) varying in the number of infusions earned by no more than 20% in two consecutive sessions; (3) making at least 70% of all responses on the AL; (4) the passage of at least 2 weeks in the FR1 phase. The FR5 training phase, usually 1–2 weeks, progressed to the extinction phase when no more than 20% variation in the number of reinforcers earned between the two sessions occurred. Mice in the saline control group were trained with the same procedure except the cocaine solution was replaced with saline. The training procedure of mice in the yoked group strictly followed that of the corresponding paired masters, except each drug delivery and presentation of visual cues were initiated by the paired masters.

### Extinction of established cocaine IVSA behavior

Mice in all three groups were trained in operant chambers with everything similar as in the acquisition phase except that all lever presses were recorded but were without programmed consequences. Subjects remained in extinction until no more than 20% variation in the number of AL responses between two consecutive sessions occurred, with a minimum of 2-week extinction period.

### Drug-primed reinstatement of cocaine seeking

Four cycles of reinstatement testing were performed in all subjects 24 h after meeting the extinction criteria (see above). In each cycle, mice were tested for their propensity to reinstate drug-seeking behavior after a challenge injection of cocaine (0, 1, 3.2 and 10 mg/kg IP, in random order) followed by at least 2-day re-extinction until they met again the extinction criteria. The *in vitro* electrophysiological studies were performed 24 h after the last extinction session. Thus, the end point of behavioral training was when the subjects were at the extinction phase. Forty-one of 43 mice reached the established criteria.

### Slice preparation and localization of MSNs in the NAc sub-regions

A schematic for *in vitro* NAc MSNs recordings is shown in the lower panel of Figure 1. Mice were decapitated, and brains were quickly removed. Coronal slices containing the NAc (300 μm thickness) were prepared with a vibratome (Leica) and incubated for at least 1 h in standard ASCF (artificial cerebrospinal fluid) composed of the following (in mM): 130 NaCl, 26 NaHCO_3_, 3 KCl, 2 MgCl_2_, 1.25 NaHPO_4_, 2 CaCl_2_ and 10 glucose [osmolality, 300 mOsm; pH 7.3-7.4, equilibrated with 95% (v/v) O_2_ and 5% (v/v) CO_2_]. All MSNs included in this study were located within the NAc, in coronal slices taken from 1.7 to 0.8 mm anterior to Bregma (Ma et al., 2012). The anterior commissure and the islands of Calleja were used as landmarks for locating the NAc core and shell sub-regions. Coordinates for recording in NAc core were 1.3–0.8 mm anterior to Bregma, within 200 μm from the edge of the anterior commissure, whereas in NAc shell coordinates were 1.7–1.0 mm anterior to Bregma, ~200–500 μm medial to the anterior commissure and ~100–800 μm dorsal to the islands of Calleja (Franklin and Paxinos, 2007).

### Electrophysiological recordings

Whole-cell patch clamp recordings of NAc MSNs were performed using methods adapted from those described previously (Cepeda et al., 1998; Cepeda et al., 2008). Cells also were identified by somatic size, basic membrane properties (input resistance, membrane capacitance and time constant), and by addition of biocytin (0.15%) to the internal solution. The patch pipette (3–5 MΩ) contained one of the following solutions (in mM): 1) *KGluc (K-gluconate internal solution)*: K-gluconate 140, Hepes 10, MgCl_2_ 2, CaCl_2_ 0.1, EGTA 1.1 and K_2_ATP 2, for voltage and current clamp; 2) *CsMeth (Cs-Methanesulfonate) internal solution*: Cs-methanesulfonate 130, CsCl 10, NaCl 4, MgCl_2_ 1, MgATP 5, EGTA 5, HEPES 10, GTP 0.5, phosphocreatine 10, leupeptin 0.1, for voltage clamp recordings (pH 7.25–7.3, osmolality, 280–290 mOsm). Access resistances were <25 MΩ. sEPSCs (spontaneous excitatory postsynaptic currents) and sIPSCs (spontaneous inhibitory postsynaptic currents) were recorded by holding the membrane at −70 mV and +10 mV, respectively, in ACSF. In specific experiments, sEPSCs were recorded in the presence of the GABA_A_ receptor antagonist, bicuculline (BIC, 20 μM) in the external solution, and by holding the membrane at −70 mV. mEPSCs (miniature excitatory postsynaptic currents) were recorded after addition of TTX (tetrodotoxin, 1 μM).

### Recordings with CsMeth internal solution

Passive membrane properties were determined in VC (voltage clamp) mode by applying a depolarizing step voltage command (10 mV) and using the membrane test function integrated in the pClamp8 software (Axon Instruments). This function reports membrane capacitance (Cm, in pF), input resistance (Rin, in MΩ) and decay time constant (Tau, in ms). This was obtained from a single exponential fit to the decay of the capacitive transient. After characterizing the basic membrane properties of the neuron, EPSCs/IPSCs were recorded for 3–6 min. The membrane current was filtered at 1 kHz and digitized at 200 μs using Clampex (Foster City, CA). Spontaneous events were analyzed off-line using the Mini Analysis Program (Jaejin Software). The Thr amplitude for the detection of a synaptic event (generally 6 pA for EPSCs and 10 pA for IPSCs), was adjusted to be 2–3 times above the root-mean-square noise level. This software was also used to calculate EPSC frequency, amplitude for each synaptic event, and to construct time-frequency histograms. Frequencies were expressed as number of events per second (Hz).

### Recordings with KGluc internal solution

Recordings started in the VC mode to measure passive membrane properties following the same procedure used with CsMeth internal solution. Then recordings were switched to current clamp (IC) mode and measurements of membrane properties were obtained following published methods (Heng et al., 2008). The RMP (resting membrane potential) was measured 5 min after the seal was ruptured. For membrane excitability assessment, the membrane potentials of all the neurons were held at −80 mV in IC mode to make the measurements from different neurons comparable. The rheobase was defined as the minimal depolarizing current necessary to evoke APs (action potentials). The Rin was calculated from the voltage response to a hyperpolarizing current pulse (−100 pA). For measurements of a single AP, a Thr was determined by differentiating the AP waveform and setting a rising rate of 10 mV/ms as the AP inflection point.

**Figure 2:**
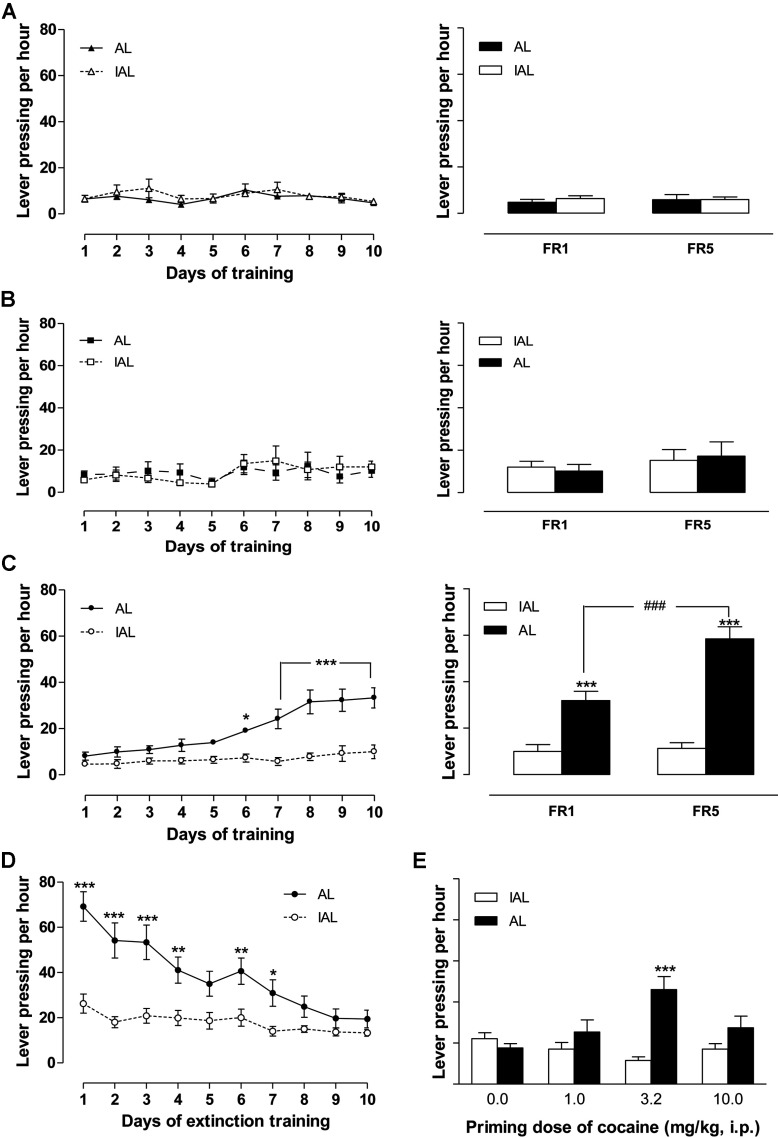
Establishment of drug-primed reinstatement of cocaine seeking (**A**–**C**) Acquisition of cocaine IVSA in saline, (**A**), yoked (**B**), and master (**C**) mice with FR1 (left) and then consolidation with FR5 (right) training schedules, *n*=13, 14, 14, respectively. (**D**) Extinguishment of the established cocaine IVSA in master mice, *n*=14. (**E**) Cocaine-primed reinstatements of drug-seeking in master mice, *n*=10–14. Blank circles or columns, IAL; solid circles or columns, AL. Data were analyzed by two-way rm-ANOVA followed by Bonferroni *post-hoc* test. *, **, ***, *P*<0.05, 0.01, 0.001, respectively, AL *versus* IAL. ^###^, *P*<0.001, FR1 *versus* FR5.

**Figure 3:**
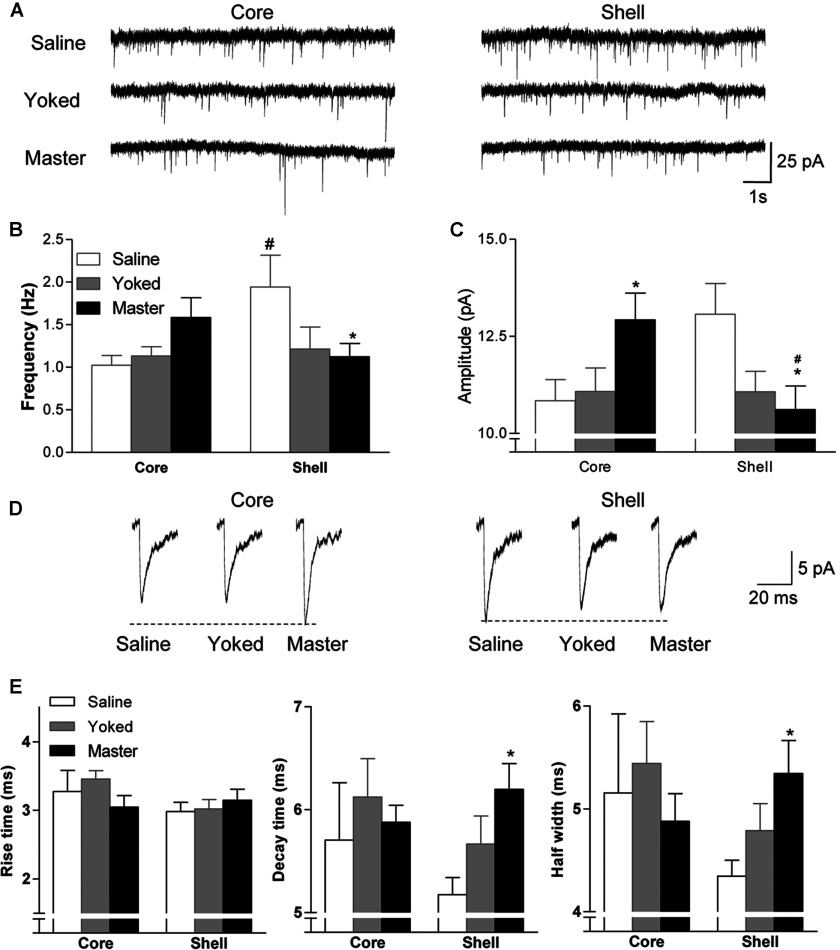
Basal activities of sEPSCs before *in vitro* cocaine treatment (**A**) Representative traces of MSN sEPSCs from NAc core/shell in the saline, yoked and master mice. Average frequency (**B**) and amplitude (**C**) of sEPSCs of MSNs in NAc core/shell from saline (blank columns), yoked (gray columns) and master (black columns) mice, number of neurons/number of mice (n/m)=10–13/6–7. (**D**) Traces represent average sEPSCs recorded in MSNs from core and shell of saline, yoked, and master mice. (**E**) Graphs indicate average values of kinetic parameters of sEPSCs in MSNs from core and shell. Decay time and half-width durations were significantly increased in cells from master mice compared with saline controls. Data were analyzed by two-way ANOVA followed by Bonferroni *post*-*hoc* test. *, *P*<0.05, compared with saline controls; #, *P*<0.05, core *versus* shell.

**Figure 4:**
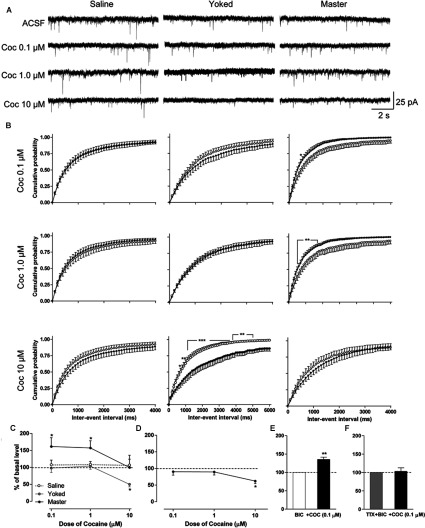
Effects of acute cocaine treatment on excitatory postsynaptic transmission of NAc MSNs (**A**) Representative traces of sEPSCs recorded in ACSF and cocaine (0.1, 1.0, and 10 μM) of NAc MSNs from saline (left), yoked (middle), and master (right) mice. (**B**). Cumulative probabilities of inter-event intervals of sEPSCs from NAc MSNs in saline (left), yoked (middle), and master (right) mice. Open and solid circles, recordings in ACSF and cocaine respectively. (**C**) Effects of acute cocaine (0.1, 1.0, and 10 μM) treatments on sEPSC frequencies. In (**B**) and (**C**), MSNs from saline, yoked, and master mice, n/m=17/7 (shell 8/6, core 9/7), n/m=11/7 (shell 5/5, core 6/5), and n/m=11/6 (shell 4/4, core 7/5), respectively. (**D**) Effects of acute cocaine (0.1, 1.0, and 10 μM) treatment on miniature EPSC frequencies. MSNs from master mice recorded in the presence of TTX (1 μM), n/m=6/4 (shell 3/3, core 3/3). (**E**) Effects of acute cocaine (0.1 μM) treatment on sEPSC frequencies of NAc MSNs from master mice in the presence of BIC (20 μM), n/m=5/4 (shell 3/2, core 2/2). (**F**) Effects of acute cocaine (0.1 μM) treatment on miniature EPSC frequencies of NAc MSNs from master mice in the presence of BIC, n/m=7/4 (shell 3/3, core 4/3). Data were analyzed by two-way (**B**, **C**) or one-way (**D**) rm-ANOVA followed by Bonferroni *post*-*hoc* test and paired Student's *t* test (**E**, **F**). *, **, ***, *P*<0.05, 0.01, 0.001, respectively, before *versus* after cocaine.

**Figure 5:**
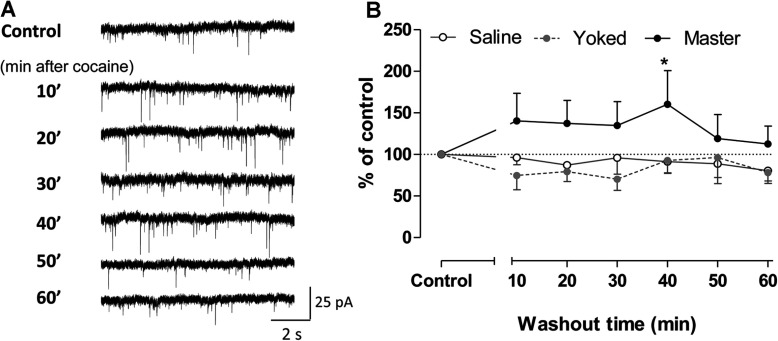
Prolonged effects of acute cocaine treatment during washout on sEPSCs of NAc MSNs (**A**) Representative traces of sEPSCs recorded in ACSF before (control) and 10–60 min after cocaine treatment. (**B**) Prolonged effects of acute cocaine treatment during washout on the average frequency of sEPSCs. Blank, gray, and dark circles, recorded from saline (n/m=14/6; shell 7/5, core 7/6), yoked (n/m=8/4; shell 4/4, core 4/3) and master (n/m=8/5; shell 4/4, core 4/3) mice. Data were analyzed by two-way rm-ANOVA followed by Bonferroni *post*-*hoc* test. *, *P*<0.05, compared with saline.

**Figure 6:**
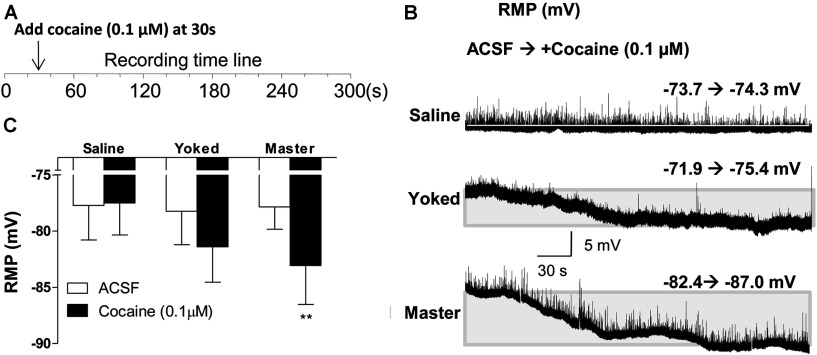
Effects of acute cocaine treatment on RMPs of NAc MSNs (**A**) Time course of cocaine treatment in IC mode. (**B**) Representative time-course of changes in RMPs recorded from saline, yoked, and master mice. (**C**) Effects of acute cocaine treatment on RMP of NAc MSNs. Open and solid bars represent RMPs in ACSF and after cocaine (0.1 μM), respectively, n/m=8/4 (shell 4/4, core 4/3). Data were analyzed by two-way rm-ANOVA followed by Bonferroni *post*-*hoc* test. **, *P*<0.01, ACSF *versus* cocaine.

**Figure 7:**
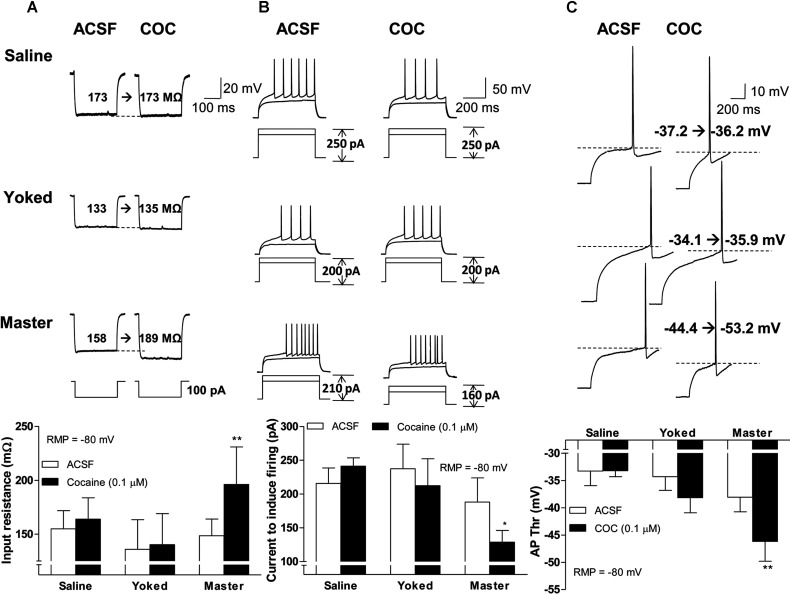
Effects of acute cocaine treatment on intrinsic excitability of NAc MSNs Effects of acute cocaine treatment on Rin (**A**), depolarizing currents necessary to evoke APs (**B**) and AP Thr (**C**) of NAc MSNs. Representative traces and graphs are shown in the upper and lower panels, respectively, n/m=8/4 (shell 4/4, core 4/3). Data were analyzed by two-way rm-ANOVA followed by Bonferroni *post*-*hoc* test. *, **, *P*<0.05, 0.01, respectively, ACSF *versus* cocaine.

Values in the figures and tables are presented as mean±S.E. of the mean. Two-way (Figures 2, 4(B) and 4(C), and Figures 5–7) or one-way (Figure 4D) rm (repeated measures) ANOVAs were followed by multiple comparisons using Bonferroni *post*-*hoc* tests, except two-way ANOVAs followed by Bonferroni *post*-*hoc* tests in Table 1 and Figure 3 and paired Student's *t* tests in Figures 4(E) and 4(F). Differences between means were considered statistically significant if *P*<0.05.

**Table 1:** Passive membrane properties (internal solution: CsMeth) of MSNs from NAc core/shell in saline, yoked and master mice in VC mode Cm, cell membrane capacitance; Rin, membrane input resistance; Tau, time constant. Data are shown as mean±S.E.M., analyzed by two-way ANOVA followed by Bonferroni *post-hoc* test.

Measurement	Sub-region	Saline	Yoked	Master
Cm (pF)	Core	92.5±12.4	82.1±4.9	87.3±6.1
	Shell	79.1±4.7	83.3±9.1	96.5±5.9
Rin (MΩ)	Core	177.2±31.2	92.6±9.0	114.3±9.8
	Shell	183.7±32.4	130.8±20.2	243.7±31.6**
Tau (ms)	Core	1.9±0.3	2.0±0.2	1.8±0.1
	Shell	1.6±0.1	1.9±0.2	2.2±0.2*

*P<0.05

**P<0.01 respectively, core *versus* shell.

## RESULTS

### Establishment of drug-primed reinstatement of cocaine-seeking behavior

Without prior operant conditioning training or food restriction, C57BL/6J mice in the master group readily acquired IVSA of cocaine (1 mg/kg per infusion) when it was reinforced on an FR1 schedule of reinforcement. The number of AL pressings was significantly higher than IAL pressings from the 6th day of training (AL *versus* IAL, *F*_1, 234_=25.43, *P*<0.0001; lever×day, *F*_9,__234_=7.79, *P*<0.0001; *post-hoc* test: at least *P*<0.05, AL *versus* IAL, Figure 2C, left). The yoked mice, trained to receive cocaine IV deliveries each time the specific paired master mice initiated IV administrations of cocaine by AL pressings, did not show any preference for the AL *versus* IAL (Figure 2B, left). Similarly, neither operant response was reinforced by programmed IV deliveries of saline in control mice, showing that the visual cue itself was not sufficient to support operant responding (Figure 2A, left). The specificity of cocaine-reinforced AL responding in master mice was more clear after transition to the FR5 schedule (FR1 *versus* FR5, *F*_1, 26_=10.81, *P*=0.0029; AL *versus* IAL, *F*_1, 26_=110.00, *P*<0.0001; lever×ratio, *F*_1,__26_=14.77, *P*=0.0007; *post*-*hoc* test, for both FR1 and FR5, *P*<0.001, AL *versus* IAL; for AL, *P*<0.001, FR1 *versus* FR5, right panel of Figure 2C). However, the change in schedule did not affect the lever pressing rates in yoked or saline groups (right panels of Figures 2A and 2B).

After a minimum 10 days of extinction training, the number of AL responses made by master mice declined to a level not different than IAL responses (days, *F*_9, 130_=7.52, *P*<0.0001; AL versus IAL, *F*_1, 26_=110.00, *P*<0.0001; lever×ratio, *F*_1, 26_=14.77, *P*=0.0007; *post*-*hoc* test, for both FR1 and FR5, *P*<0.001, AL *versus* IAL; for AL, *P*<0.001), suggesting that drug-seeking behavior was extinguished by this procedure (Figure 2D). The drug-primed reinstatement tests in master mice showed that the number of AL responses was significantly higher than that of IAL responses when an IP injection of cocaine at 3.2 mg/kg, but not at 1 or 10 mg/kg, was given before reinstatement testing (dose×lever, *F*_1, 45_=8.97, *P*<0.0001; *post-hoc* test, *P*<0.001, AL *versus* IAL, primed by cocaine at 3.2 mg/kg, Figure 2E). This inverted U-dose effect is consistent with past studies (Weissenborn et al., 1996; Rosenzweig-Lipson et al., 1997). The lever pressing paradigm in the yoked and saline controls was not changed by an IP priming injection of cocaine (0, 1, 3.2 and 10 mg/kg, results not shown).

### Membrane and synaptic properties of NAc MSNs *in vitro* before acute cocaine re-exposure

Electrophysiological studies in slices from trained mice were performed 24 h after the last extinction session. MSNs in NAc shell from master mice showed significant increases in membrane Rin compared with cells in the core compartment measured in VC mode (sub-region, F_1, 60_=6.04, *P*=0.0169; behavioral procedure, F_2, 60_=3.64, *P*=0.0321; *post-hoc* test, *P*<0.01, shell *versus* core MSNs from master mice, Table 1). Higher Rin in the shell of master mice suggested that MSNs in this region could be more excitable.

Compared with saline controls, MSNs in the NAc core of masters showed a significantly higher frequency and larger amplitude of sEPSCs, whereas in the NAc shell, a lower frequency and smaller amplitude of sEPSCs were observed (sub-region×behavioral procedure, for frequency, *F*_2, 63_=4.53, *P*=0.0145; for amplitude, *F*_2, 63_=6.43, *P*=0.0029; *post*-*hoc* test, for both frequency and amplitude, *P*<0.05, master *versus* saline in the MSNs of NAc core and shell). This up-regulation of sEPSCs in the core was specifically related to active, contingent drug use/extinction history as it did not occur in yoked mice or saline controls. Interestingly, while the frequency of sEPSCs in MSNs of the core region in yoked mice was similar to that of the saline group, in the shell it was similar to the master group, suggesting that down-regulation of sEPSCs in the shell is the consequence of repeated exposure to cocaine, either in a contingent or non-contingent manner (Figures 3A–3C). Furthermore, the decay time and duration at half-width of sEPCS of shell MSNs were longer in master mice than in saline controls (sub-region×behavioral procedure, for decay time, *F*_2, 63_=3.21, *P*=0.0470; for half-width duration, *F*_2, 63_=3.35, *P*=0.0415; *post*-*hoc* test, *P*<0.05, master *versus* saline in the MSNs of NAc core and shell for both frequency and amplitude).

Changes in sIPSCs were observed as well. The sub-regional bidirectional modification in the frequency of synaptic activity, decreased in MSNs from the shell but increased in MSNs from the core compartment, was also observed. However, the amplitude of sIPSCs remained unchanged in master mice compared with saline controls (results not shown).

### Response of MSNs in the NAc after re-exposure to cocaine *in vitro*

#### Effects on synaptic transmission during and after cocaine bath application

MSNs from master, yoked and saline control mice showed dose-dependent responses to acute *in vitro* cocaine application (*F*_2, 105_=3.30, *P*=0.0406) that were related to the specific behavioral procedure (*F*_2, 105_=4.97, *P*=0.0086, Figure 4C). Thus, NAc MSNs from saline controls showed no response to increasing concentrations (0.1, 1.0 and 10 μM) of cocaine (left panels of Figures 4A and 4B). In contrast, the average sEPSC frequency in MSNs from master mice was increased during application of cocaine at 0.1 and 1.0 μM, but decreased to control levels at 10 μM (right panels of Figures 4A and 4B). The average frequency of sEPSCs in MSNs from yoked mice showed a response only to the high dose of cocaine and this effect was inhibitory (middle panels of Figures 4A and 4B). This dose-dependent response of NAc MSNs did not show sub-regional differences and data from shell and core were pooled together. The number of cells from each compartment was roughly half and half. Additional experiments demonstrated that the modification of sEPSCs frequency in master mice by low doses of cocaine (0.1, 1.0 μM) was abolished in the presence of TTX (*F*_3, 5_=7.326, *P*=0.0030; *post*-*hoc* test, *P*<0.05, cocaine at 10 *versus* 0, 0.1, 1 μM, Figure 4D). Furthermore, after pharmacological isolation of sEPSCs by addition of BIC to the perfusate, the average frequency of sEPSCs in master mice was also increased by cocaine at 0.1 μM (*t*_4_=5.670, *P*=0.0048, Figure 4E), and this up-regulation disappeared in the presence of TTX (*t*_6_=0.3178, *P*=0.7614, Figure 4F). No changes of sEPSCs amplitude were observed in master, yoked or saline control mice, suggesting an AP-dependent mechanism of changes occurring in response to acute cocaine in NAc MSNs from mice subject to different behavioral procedures. In addition, the sIPSCs of NAc MSNs from mice in all groups did not show any specific modifications by cocaine bath application (0.1, 1.0, 10 μM) (results not shown). These results suggest that long-distance, excitatory projections, but not local inhibitory inputs, to NAc MSNs of master mice are involved in mediating the acute response of cocaine.

Interestingly, after an initial depressing effect during application of the high cocaine dose (10 μM), cells from master mice reverted to an increased level of sEPSC frequency shortly after washout, i.e., the frequency was higher than before cocaine application. This effect persisted for more than 40 min (*F*_2, 189_=10.97, *P*<0.0001; *post*-*hoc* test, *P*<0.05, master *versus* saline at 40 min of cocaine washout, Figure 5B) suggesting a sort of rebound synaptic excitation, as well as the existence of persistent changes in synaptic activity of NAc neurons after re-exposure to cocaine. This effect did not occur in cells from saline or yoked mice, as the frequency of sEPCSs was similar before cocaine and after washout. In contrast to sEPSCs, no changes in the frequency of sIPSCs were detected after acute cocaine re-exposure *in vitro*. This suggests sensitization of synaptic activity after acute cocaine only involved glutamatergic inputs.

#### Effects of low-dose cocaine re-exposure on cell membrane properties and excitability

Recordings in IC mode (KGluc as the internal solution) demonstrated no differences in RMPs of NAc MSNs among saline, yoked and master mice before cocaine re-exposure *in vitro*. However, MSNs from master, but not saline or yoked mice, were significantly hyperpolarized by a 0.1 μM cocaine application (cocaine dose×behavioral procedure, *F*_2, 21_=3.74, *P*=0.0408; *post*-*hoc* test, *P*<0.01, cocaine 0 *versus* 0.1 μM in master mice, Figure 6). In contrast, the intrinsic excitability of MSNs from master mice was increased as evidenced by significantly higher Rin (cocaine -/+, *F*_1, 21_=6.15, *P*=0.0217; *post*-*hoc* test, *P*<0.01, cocaine 0 *versus* 0.1 μM in master mice, Figure 7A), lower depolarizing currents necessary to evoke APs (all measured at a fixed membrane potential of −80 mV) (cocaine×behavioral procedure, *F*_2, 21_=4.17, *P*=0.0298; *post*-*hoc* test, *P*<0.05, cocaine 0 *versus* 0.1 μM in master mice, Figure 7B), and decreased Thr for AP firing (cocaine×behavioral procedure, *F*_2, 21_=3.48, *P*=0.0498; *post*-*hoc* test, *P*<0.01, cocaine 0 *versus* 0.1 μM in master mice, Figure 7C). The amplitude of the AP AHP (afterhyperpolarization) in MSNs from master mice was not changed after cocaine application [−9.92±0.80 in ACSF and −8.74±0.97 after cocaine (t_6_=1.405, *P*=0.21)]. Similarly, no significant differences of AHP were detected in saline or yoked mice.

## DISCUSSION

This study used a complex behavioral training procedure that better replicates the experience of voluntary drug intake, followed by relapse, commonly seen in the human condition, although it is worth noting that most addicts experience abstinence, but not extinction learning. Our procedure included acquisition, extinction and repeated reinstatement/extinction cycles in mice trained by contingent cocaine IVSA. With this multi-staged paradigm it was possible to explore functional alterations of NAc MSNs using *in vitro* electrophysiological recordings in slices before and after acute re-exposure to cocaine. Our data clearly demonstrate differences in passive and active membrane properties of MSNs, as well as synaptic adaptations before and after acute cocaine application in slices from saline, yoked and master mice. Some neuroadaptations were region-specific, while others were observed in both compartments. In master mice, MSNs displayed higher Rin in the shell of NAc under basal conditions, i.e., before acute re-exposure to cocaine, compared to yoked and saline controls. In addition, cells from master mice showed an AP-dependent increase in sEPSC frequency after re-exposure to a low dose of cocaine *in vitro*. Acute cocaine also induced membrane hyperpolarization, but concomitantly increased membrane excitability of MSNs from master mice, as evidenced by increased Rin, decreased current required to induce firing and hyperpolarized AP Thr. These changes were not observed in yoked mice or saline controls. To the best of our knowledge, this is the first report of functional alterations of NAc MSNs after chronic contingent *versus* non-contingent cocaine exposure using IVSA in mice, and also the first to examine the effects of extinction training in conjunction with an *in vitro* equivalent of cocaine-primed drug-seeking behavior at the cellular and synaptic levels.

### Regional and non-regional differences in NAc MSNs before and after acute cocaine application in slices

Passive membrane properties and spontaneous synaptic transmission of MSNs from master mice were different in the NAc shell *versus* core before cocaine re-exposure, consistent with previous observations using non-contingent administration (Martin et al., 2006), in which opposite changes in excitability of MSNs were reported between shell and core. The decrease in frequency and amplitude of spontaneous synaptic activity in the shell occurred in conjunction with increases in membrane input resistance. While this may appear paradoxical, this increase could represent another synaptic adaptation in response to reduced glutamatergic input, a sort of synaptic scaling (Turrigiano and Nelson, 2004). For example, in NAc neurons it has been shown that postsynaptic membranes are capable of adjusting excitability in response to basal shifts in excitatory synaptic input, a phenomenon called homeostatic synapse-driven membrane plasticity (Ishikawa et al., 2009). In contrast to our findings, another study reported increased frequency and amplitude of mEPSCs in the shell compartment (Kourrich et al., 2007). However, in this study very young mice were used and the cocaine treatment was non-contingent. The apparent discrepancy with our results underlines that age and mode of administration play a critical role in defining the outcomes of electrophysiological changes.

There was no difference between core and shell in response to acute cocaine treatment. The prolonged effects of acute treatment during cocaine washout were also homogeneous in both compartments. Moreover, acute cocaine treatment-induced changes in the intrinsic cellular excitability, including RMP, Rin, current necessary to induce firing and Thr for APs also did not show sub-regional differences. These results suggest that although cocaine IVSA mice show sub-regional differences in passive membrane properties, the increased membrane input resistance of NAc shell MSNs from master mice may not be responsible for mediating the cocaine-primed drug-seeking behavior but a result of the training or extinction procedure. The possible mechanism of this change is presently unknown but may involve modifications in DA and second messenger cascades that alter specific K^+^ conductances (Dong et al., 2004; Dong et al., 2005). It is also possible that DA levels play a role. For example, withdrawal from cocaine self-administration decreases tyrosine hydroxylase levels in NAc shell but not core (Self et al., 2004). The absence of sub-regional differences in response to cocaine re-exposure of MSNs in the core *versus* shell is consistent with previous *in vivo* studies which showed no sub-regional difference in the NAc in reinstatement of cocaine-seeking (Famous et al., 2007).

### Excitatory but not inhibitory synaptic transmission of NAc MSNs was involved in specific responses to cocaine re-exposure in master mice

Our results showed that both sEPSCs and sIPSCs were modulated by the chronic cocaine exposure history. Under basal conditions, i.e., before cocaine application in slices, NAc MSNs from master mice showed lower frequency and smaller EPSC amplitude in the shell, but significantly higher frequency and bigger amplitude in the core. Similar changes in sIPSCs frequency, but not amplitude, were observed. However, changes to acute cocaine re-exposure *in vitro* were only detected in the frequency of sEPSCs, but not sIPSCs. In addition, the alterations in the frequency of the sEPSCs from master mice was AP-dependent, suggesting adaptations in excitatory projections from pre-/infra-limbic cortex, but not of local inhibitory, GABAergic inputs, mediated cocaine-primed reinstatement of drug-seeking. This is consistent with accumulating evidence that glutamate inputs are necessary for drug-primed cocaine-seeking behaviors, which could be blocked by ablating (Peters et al., 2008) or manipulating the cortical-NAc glutamatergic projections either pharmacologically (Berglind et al., 2009) or optogenetically (Suska et al., 2013). While an essential role for cortical inputs to NAc has been acknowledged, other excitatory inputs can induce plastic changes in NAc neurons. In particular, a strong and specific projection from the ventral hippocampus to the medial NAc shell has been shown to enhance synaptic strength after cocaine administration, albeit using a non-contingent administration paradigm (Britt et al., 2012). As MSNs in both compartments were sensitized by acute re-exposure to cocaine in our IVSA paradigm, it is likely that inputs from the prefrontal cortex and basolateral amygdala also play an important role. Although changes in NAc GABA_B_ receptor-mediated activity *in vitro* from rats trained by either contingent or non-contingent cocaine have been reported (Mu et al., 2010; Wolf, 2010), a few studies have focused directly on the involvement of GABAergic mechanisms in cocaine-primed reinstatement of drug-seeking, except for one study showing that administration of GVG, an irreversible GABA (γ-aminobutyric acid) transaminase inhibitor, dose-dependently inhibits cocaine-induced reinstatement of drug-seeking behavior in rats (Peng et al., 2008).

### Sensitized synaptic activity was observed in NAc MSNs from master, but not yoked or saline mice

Application of low doses of cocaine in slices (0.1, 1 μM) increased the frequency of sEPSCs in master, but not saline or yoked mice, suggesting sensitized glutamate transmission in the NAc MSNs. These findings are in agreement with results in dorsal striatum using chronic administration of metamphetamine (Bamford et al., 2008). A prolonged state of corticostriatal depression is followed by a paradoxical pre-synaptic potentiation upon re-exposure to the drug. Because adding BIC had no effects on the response of sEPSCs to a low dose of cocaine, local inhibitory circuits seemed not involved. However, this sensitized response in master mice was abolished in the presence of TTX, suggesting that an AP-dependent glutamate release was sensitized at the pre-synaptic level and could be involved in mediating the cocaine-primed drug-seeking behaviors. Although a tight relationship between glutamate levels and cocaine-primed reinstatement has been demonstrated in rats by *in vivo* cocaine IVSA (Baker et al., 2003; Madayag et al., 2007), lack of examination of glutamate synaptic activity in the yoked subjects limits the relevance of these observations. Our current data demonstrated that (1) increased AP-dependent release of glutamate might be involved in cocaine seeking in the mouse IVSA model; and (2) this sensitized glutamate synaptic activity probably resulted from the active drug-taking history, not the prolonged effects of chronic cocaine exposure history itself as it did not occur in yoked mice. An additional mechanism that could contribute to sensitized responses to cocaine is the well-known enhancing effect of DA, *via* D1 receptors, on glutamatergic activity (Cepeda et al., 1993; Ma et al., 2009), as re-exposure to cocaine produces an increase of DA concentration in slices (Ortiz et al., 2010).

An interesting finding is that in the presence of TTX sEPSCs frequency of NAc MSNs from master mice showed a similar response to cocaine as that from yoked mice, i.e., no response to low doses (0.1, 1 μM), whereas frequency was decreased by a high dose (10 μM) of cocaine. Thus, we hypothesize that pharmacological effects of chronic IV cocaine administration after prolonged withdrawal is encoded in the NAc MSNs by an AP-independent mechanism, possibly mediated by postsynaptic mechanisms. However, the long persistence of compulsive drug-seeking behaviors was mediated by an AP-dependent mechanism, which more possibly resulted from alterations in pre-synaptic pathways. This AP-dependent mechanism showed a glutamate sensitized response to low dose (0.1, 1 μM) cocaine treatments, but the AP-independent response was only observed at the high dose (10 μM). It appears that pre- and postsynaptic mechanisms in the NAc play differential roles in the long-persistence of emotional/motivational and pharmacological-related effects, respectively. Indeed, it has been demonstrated that the down-regulation of sEPSCs in striatal MSNs occurs in response to a high dose of cocaine (10 μM) (Wu et al., 2007). However, at high concentrations cocaine may produce anesthetic effects by reducing the amplitude of Na^+^ currents (Crumb and Clarkson, 1990).

Could changes in intrinsic excitability of NAc MSNs after cocaine re-exposure be attributable to contingent *versus* non-contingent cocaine administration, or route of administration? Our data demonstrated that after a prolonged period of drug withdrawal, no differences occurred in the basal values of RMP, Rin, current required to induce APs and Thr for firing from mice with IVSA cocaine history *versus* saline controls. This is consistent with the only previous study using contingent IV cocaine administration in rats, although in this study a temporary decrease of MSN intrinsic excitability during the early stage of cocaine withdrawal was observed (Mu et al., 2010). However, there is evidence that during both early (within 1 week) and late stage (after 2–3 weeks) of withdrawal from repeated non-contingent IP injections of cocaine significant decreases of intrinsic excitability of NAc MSNs occur (Kourrich and Thomas, 2009; Mu et al., 2010). More importantly, the NAc MSNs from IV yoked mice, which serves as a better non-contingent administration control for IV masters than mice receiving non-contingent IP injections, did not show any changes in RMP, Rin, depolarizing current necessary to evoke APs, and Thr of APs, which is different from the controls with non-contingent IP injections. This suggests that the intrinsic excitability of NAc MSNs, although clearly related to the stage of withdrawal, does not depend on contingent or non-contingent cocaine administration. The way by which cocaine is delivered, IP or IV, is hypothesized to be responsible for alterations of intrinsic excitability of NAc MSNs in mice with a history of chronic cocaine exposure. For example, in Mu's study, after a 3-week cocaine withdrawal the membrane excitability of NAc MSNs, which remained low in IP treated rats, returned to a normal level in IVSA-treated rats. This can be explained by different pharmacokinetics of these two routes of drug administration. The behavioral effects of IV administration of cocaine has a more rapid onset and termination relative to IP administration (O’Dell et al., 1996). In humans, IV administration of cocaine produces greater physiological changes and more intense subjective effects relative to other routes (Resnick et al., 1977).

Although the present methodology probably represents the best way to examine changes in NAc MSN properties in a mouse model of drug-seeking behavior, one limitation is that, because of the complex training paradigm, it is difficult to determine the exact source and timing of electrophysiological changes in NAc MSNs in the *in vitro* studies. Thus, permanent changes could be attributed to acquisition, extinction, and/or reinstatement phases. Notwithstanding this limitation, our study opens new alternatives for a deeper understanding of the mechanisms of drug-seeking behavior. The availability of genetically modified laboratory animals, e.g., mice expressing green fluorescent protein as a reporter of specific neuronal populations of the striatum, will allow defining better the contribution of the direct and indirect pathways in drug addiction. Based on our previous work (Cepeda et al., 2008; Ma et al., 2012) as well as the current literature, we can speculate that sensitized responses to cocaine occur mainly in D1 receptor-containing MSNs (see also (Lobo et al., 2010; Pascoli et al., 2010; Lobo and Nestler, 2011; Chandra et al., 2013; Smith et al., 2013). For example, it has been shown that overexpression of the transcription factor ΔFosB in direct, but not indirect, pathway MSNs enhances behavioral responses to cocaine (Grueter et al., 2013), and ERK (extracellular signal-regulated kinase) phosphorylation after acute or chronic cocaine injections is confined to D1 MSNs in NAc and dorsal striatum (Bertran-Gonzalez et al., 2008). In addition, a selective increase in spine density occurred in D1 MSNs after chronic cocaine exposure (Kim et al., 2011). However, a role for D2 MSNs cannot be ruled out (Lobo et al., 2010) although, for the most part, adaptations in D2 MSNs generally oppose addictive behaviors (Lobo and Nestler, 2011).

## CONCLUSIONS

Using a complex paradigm of cocaine use consisting of repeated cycles of extinction/reinstatement, a model that closely resembles the human condition, we demonstrate electrophysiological neuroadaptations in NAc MSNs due to either contingent (IVSA in master mice) or non-contingent (yoked mice) administration. Changes after a long withdrawal period were compartment specific while those occurring after acute re-exposure to cocaine occurred in both core and shell. We also demonstrate sensitized glutamate transmission of NAc MSNs from master mice in response to acute cocaine application and suggest a promising strategy to counter drug-primed cocaine-seeking. Disruption of glutamatergic inputs onto MSNs or blockade of glutamate receptors in NAc could prevent relapse induced by drugs that have been previously abused.
